# Medico-legal Knowledge and Awareness Among Anaesthesiology and Reanimation Physicians in Türkiye: A Nationwide Survey

**DOI:** 10.4274/TJAR.2026.262588

**Published:** 2026-10-30

**Authors:** Hacı Yusuf Güneş

**Affiliations:** 1 Van Yüzüncü Yıl University Faculty of Medicine Department of Anaesthesiology and Reanimation, Van, Türkiye

**Keywords:** Anaesthesiology, duties and authority, informed consent, medical liability, professional responsibility, health knowledge and practice

## Abstract

**Objective:**

Anaesthesiologists undertake substantial clinical, organisational, and medico-legal responsibilities throughout perioperative care. This nationwide survey evaluated self-reported and questionnaire-assessed knowledge of professional, organisational, and medico-legal responsibilities among anaesthesiology and reanimation physicians practising in Türkiye.

**Methods:**

This nationwide cross-sectional survey included anaesthesiology and reanimation physicians practising in Türkiye. Participants completed a structured 36-item questionnaire assessing professional duties, legal responsibilities, informed consent, documentation, and the medico-legal framework. A total of 311 physicians participated. Descriptive statistics and exploratory subgroup analyses were performed.

**Results:**

Participants demonstrated excellent awareness of core clinical responsibilities, with 98-99% correctly identifying essential duties such as preoperative assessment, anaesthetic technique selection, and perioperative decision-making. However, important knowledge gaps were identified in the organisational and medico-legal domains. Approximately half of the participants were unaware of responsibilities related to operating theatre management and sterilisation processes, and only 47% correctly recognised responsibility for anaesthesia-related equipment and supplies. Although only 43% reported adequate knowledge of current legal regulations, knowledge assessed by the questionnaire varied considerably across domains. Nearly half of the participants had not previously taken part in structured medico-legal educational activities.

**Conclusion:**

Despite excellent awareness of core clinical responsibilities, important deficiencies were identified in questionnaire-assessed medico-legal knowledge, while self-perceived knowledge of current legal regulations remained limited. Structured medico-legal education should be integrated into residency training and continuing professional development to strengthen professional accountability, documentation practices, physician-patient communication, and safe anaesthesiology practices.

**Main Points**

• This nationwide survey demonstrates that anaesthesiology and reanimation physicians in Türkiye have excellent awareness of core clinical responsibilities, but exhibit important deficiencies in organisational and medico-legal responsibilities.

• Knowledge gaps were particularly evident regarding operating room management, sterilisation processes, responsibilities for anaesthesia-related equipment, and legal regulations.

• Participants’ self-perceived medico-legal knowledge did not consistently correspond with their questionnaire-assessed knowledge across several medico-legal domains.

• The findings support the integration of structured medico-legal education, documentation standards, and informed consent training into residency curricula and continuing professional development programmes.

• Strengthening medico-legal literacy is essential for promoting professional accountability, effective physician-patient communication, and safe anaesthesiology practice.

## Introduction

Physicians assume substantial clinical, ethical, and legal responsibilities in the delivery of healthcare. Beyond providing medical treatment, they are expected to practise in accordance with professional standards, respect patient autonomy, safeguard confidentiality, and comply with the statutory and regulatory framework governing medical practice. Consequently, medical malpractice and professional liability have become increasingly important challenges in contemporary healthcare systems, with significant implications for both physicians and patients.^1, 2, 3^ Malpractice claims may arise when clinical practice deviates from accepted standards of care or when legally protected patient rights are compromised.^4, 5, 6^

The physician-patient relationship extends beyond clinical care and encompasses ethical as well as legal responsibilities. In many legal systems, including Türkiye, physicians are expected to exercise an appropriate standard of care while fulfilling their obligations regarding informed consent, confidentiality, documentation, and professional accountability.^7, 8^

Limited knowledge of professional responsibilities may increase medico-legal risk and negatively affect both physician behavior and patient safety. Despite increasing medico-legal complexity in contemporary healthcare systems, physicians’ knowledge of legal responsibilities and regulatory obligations remains variable, and structured medico-legal education is not routinely incorporated into undergraduate or postgraduate medical training in many healthcare settings. Accordingly, strengthening physicians’ medico-legal literacy has become increasingly important, not only for mitigating malpractice risk but also for reinforcing professional accountability, improving documentation practices, enhancing physician-patient communication, and supporting patient-centred care. These educational priorities have long been recognised in the medical literature, where greater integration of legal principles into undergraduate and postgraduate medical training has been advocated.^9, 10, 11, 12, 13^

Anaesthesiology represents a particularly demanding specialty in which complex clinical decision-making, perioperative risk management, interdisciplinary communication, and high-quality documentation intersect in a high-risk environment. Although remarkable advances in monitoring technologies, patient safety initiatives, and perioperative standards have substantially reduced anaesthesia-related morbidity and mortality over recent decades, medico-legal challenges remain an important aspect of contemporary anaesthesiology practice.^14, 15, 16^ Medication errors, perioperative adverse events, communication failures, documentation deficiencies, and disputes related to informed consent remain among the leading causes of medico-legal claims in anaesthesiology practice. Accordingly, comprehensive knowledge of professional responsibilities, legal obligations, and applicable regulatory frameworks represents an essential component of contemporary anaesthesiology practice.^17, 18, 19, 20, 21, 22^

In Türkiye, physicians’ professional and medico-legal responsibilities are governed by a comprehensive legal framework comprising constitutional provisions, statutory legislation, and healthcare-specific regulations. The Constitution of the Republic of Türkiye, the Regulation on the Operation of Inpatient Treatment Institutions, the Fundamental Law on Healthcare Services, the Patient Rights Regulation, the Turkish Penal Code, and the Turkish Code of Obligations define physicians’ obligations regarding professional conduct, informed consent, documentation, confidentiality, and legal accountability. Nevertheless, physicians’ awareness and understanding of these legal responsibilities may vary considerably according to their education, training, and professional experience.^23, 24, 25, 26, 27, 28^

To our knowledge, this is the first nationwide study designed to systematically evaluate professional and medico-legal knowledge and awareness among anaesthesiology and reanimation physicians in Türkiye across multiple domains, including statutory professional duties, institutional responsibilities, informed consent, documentation obligations, and relevant legal regulations. Unlike previous studies that focused solely on malpractice or legal regulations, the present study provides the first multidimensional evaluation of physicians’ professional responsibilities and medico-legal awareness. Accordingly, the primary objective of this study was to evaluate both self-reported and questionnaire-assessed medico-legal knowledge among anaesthesiology and reanimation physicians practising in Türkiye and to identify knowledge gaps that may inform future educational strategies and healthcare policy.

## Methods

This cross-sectional survey was approved by the Non-Interventional Clinical Research Ethics Committee of Van Yüzüncü Yıl University (decision no: 2023/13-10, date: December 8, 2023) and conducted in accordance with the ethical principles of the Declaration of Helsinki.

### Study Population

A nationwide cross-sectional survey was disseminated electronically to anaesthesiology and reanimation physicians actively practising in Türkiye via the Turkish Society of Anaesthesiology and Reanimation (TARD) mailing lists and professional whatsapp groups. Participation was voluntary, anonymous, and uncompensated. The survey employed convenience and snowball sampling through professional networks. Because the exact number of physicians who received or viewed the questionnaire could not be determined, the response rate could not be calculated.

### Questionnaire Development

The questionnaire was specifically designed for the present study by the corresponding author to evaluate anaesthesiology and reanimation physicians’ knowledge and awareness of professional responsibilities, medico-legal obligations, informed consent, medical documentation, and the legal framework governing medical practice in Türkiye. Questionnaire development was guided by a comprehensive review of the Turkish medico-legal framework governing medical practice. In addition, current national and international literature in anaesthesiology addressing medical liability, informed consent, documentation, and medico-legal responsibilities was reviewed to ensure comprehensive coverage of the key knowledge domains.

The draft questionnaire was reviewed by senior anaesthesiology and reanimation specialists who have extensive expertise in health law and considerable experience in medico-legal practice. The reviewers evaluated the clarity, relevance, comprehensiveness, and legal accuracy of each questionnaire item. Their recommendations were incorporated into the final version to improve clarity, minimise ambiguity, and ensure legal accuracy.

The questionnaire was designed to assess factual knowledge of the current Turkish medico-legal framework rather than attitudes or latent psychological constructs. For dichotomous and three-option knowledge items (e.g., True/False/I do not know or Yes/No/I do not know), the legally correct response was determined a priori according to the explicit wording and legal definitions contained in the applicable legislation and regulations in force at the time of the study. For multiple-response items, all legally correct options were predefined based on the relevant legislation, and participants’ awareness was evaluated based on the number of correctly identified responses. Because the questionnaire assessed factual knowledge of current legislation rather than latent constructs, formal psychometric validation was not performed. No pilot testing was undertaken before nationwide dissemination; this has been acknowledged as a limitation of the study. The complete questionnaire, scoring principles, and response distributions for all 36 survey items are provided in Supplementary Table S1 to ensure transparency and reproducibility. Because question 26 addressed a presumed minimum timeframe of 24 hours for obtaining informed consent, this item was re-examined in light of the applicable legal framework during the revision process. Article 18 of the Turkish Patient Rights Regulation stipulates that, except in emergencies, information should be provided to the patient, and the patient should be allowed a reasonable period for consideration; it does not establish a universally applicable 24-hour minimum timeframe. Therefore, Question 26 was retained in the supplementary questionnaire for transparency but was excluded from the interpretation of questionnaire-assessed factual knowledge.

The survey was created using google forms and distributed electronically to members of the TARD via e-mail. The survey link was also disseminated through professional whatsapp groups comprising anaesthesiology and reanimation physicians practicing in Türkiye. Before accessing the questionnaire, participants were required to confirm their willingness to participate by selecting the statement “I agree to participate,” which was accepted as the provision of electronic informed consent.

### Survey Instrument

The survey instrument comprised 36 items organised into four predefined domains: (1) demographic characteristics (6 items), (2) duties and professional responsibilities of anaesthesiologists (8 items), (3) legal responsibilities and applicable laws and regulations (6 items), and (4) obligations related to patient information, informed consent, and medical documentation (16 items). The completion time was approximately five minutes. Although the survey was anonymous and direct personal identifiers were not collected, the dataset was reviewed for potential duplicate submissions based on demographic characteristics and response patterns before analysis (Table 1).

### Sample Size Calculation

The minimum required sample size was estimated using SurveyMonkey’s sample size calculator, assuming a finite population of approximately 11,000 anaesthesiology and reanimation physicians in Türkiye. The calculation was intended to ensure adequate precision for descriptive estimates rather than statistical power for subgroup comparisons. Assuming a 90% confidence level and a 5% margin of error, the minimum required sample size was estimated to be 266 participants. The sample size calculation was performed to provide adequate precision for descriptive estimation of survey responses and was not specifically powered for hypothesis testing or subgroup comparisons. Accordingly, all subgroup analyses should be interpreted as exploratory. A final analysis was conducted on the remaining 311 valid questionnaires.

### Statistical Analysis

Statistical analyses were performed using SPSS version 20.0 (IBM Corp., Armonk, NY, USA). Descriptive statistics were expressed as mean ± standard deviation for continuous variables and as frequency (n) and percentage (%) for categorical variables. Associations between categorical variables were assessed using Pearson’s chi-square test or Fisher’s exact test, as appropriate. A two-sided *P* value was considered statistically significant. Subgroup analyses were conducted to explore associations between participant characteristics and medico-legal knowledge; however, these analyses were considered exploratory because the study was not specifically powered for comparisons between subgroups. For descriptive analyses, responses to Likert-scale items were summarised according to the five original response categories. Where appropriate, adjacent categories (strongly agree/agree and strongly disagree/disagree) were combined to facilitate interpretation, as specified in the results section. For multiple-response questions, percentages were calculated according to the total number of participants, and respondents were permitted to select more than one option where applicable.

## Results

A total of 311 anaesthesiology and reanimation physicians completed the survey. Overall, participants demonstrated excellent awareness of core clinical responsibilities but had substantially lower knowledge across several organisational and medico-legal domains. Associations with professional experience differed across individual questionnaire items.

### Participant Characteristics

A total of 311 participants completed the questionnaire. Of these, 59.2% were male and 40.8% were female. Specialist physicians (52.7%) and residents (28.3%) constituted over four-fifths of the study population. Regarding professional experience, 32.2% had 11-20 years, 23.8% had 0-5 years, 27.0% had 6-10 years, and 17.0% had more than 20 years of experience. The largest proportion of participants worked in state university hospitals (38.6%) (Table 2). The complete distribution of responses for all 36 questionnaire items is provided in Supplementary Table S1 to ensure comprehensive reporting and transparency.

### Knowledge of Duties and Authority of the Anaesthesiologist

Participants demonstrated consistently high levels of awareness regarding their core clinical responsibilities (Figure 1). Correct response rates ranged from 84% to 99%, with the highest levels observed for preoperative evaluation, operating room scheduling, selection of anaesthetic technique, and perioperative decision-making (98-99%).

Nevertheless, important knowledge gaps remained regarding operating theatre management, sterilisation processes, documentation responsibilities, and the management of anaesthesia-related equipment and supplies. Awareness of documentation-related legal responsibilities was also suboptimal, with only 67.2% of participants correctly recognising the legal implications of failing to maintain medical records (Figure 2).

### Medico-legal Awareness and Determinants

Medico-legal awareness varied considerably across the domains evaluated (Figure 3). Only 43% of participants self-reported adequate knowledge of current legal regulations (strongly agree and agree) (Figure 3A). Participation in structured medico-legal educational activities remained limited, with 46% of respondents reporting no prior attendance at relevant scientific meetings (Figure 3B). Notably, 40% of anaesthesiologists self-reported prior exposure to legal complaints or judicial investigations (Figure 3C). Encouragingly, a substantial majority (94.9%) expressed a willingness to participate in future training programs. Awareness of disciplinary sanctions under Article 657 of the Civil Servants Law remained high (87%), although some uncertainty persisted (Figure 3D).

To further explore factors associated with medico-legal exposure, chi-square analyses were performed. No statistically significant association was found between gender and exposure to legal complaints (*P*=0.113). In contrast, a significant association was observed between professional experience and exposure to legal complaints (*P* < 0.001), with the likelihood of legal complaints increasing with years of practice. Similarly, academic rank was significantly associated with legal complaint exposure (*P*=0.001), with higher academic rank associated with increased reporting of prior legal complaints. While exposure to legal complaints was independent of gender, its significant association with professional experience likely reflects cumulative medico-legal exposure over time rather than differences in individual risk.

### Knowledge of Medico-legal Principles, Documentation, and Legal Framework

Participants demonstrated variable levels of knowledge across different medico-legal domains (Figure 4). Regarding informed disclosure, 84.6% of participants correctly distinguished informed disclosure from informed consent, and 84.2% correctly identified the concept of informed disclosure. Almost all participants (96.8%) correctly defined informed consent. However, knowledge regarding several practical legal principles was considerably lower. Only 39.5% correctly recognised that informed consent remains valid throughout the entire course of treatment, 49.5% correctly identified the patient’s right to withdraw consent, and 43.1% correctly recognised the burden of proof in informed consent-related disputes. Knowledge of documentation responsibilities also varied. Most participants recognised that corrections to anaesthesia records must remain legible (88.1%) and nearly all recognised document forgery as a criminal offence (94.9%). However, only 56.6% correctly identified the minimum legal retention period for anaesthesia records, whereas awareness of the legal ownership of medical records was substantially lower. The complete response distribution for all questionnaire items is presented in Supplementary Table S1.

Self-reported knowledge of legal regulations varied significantly by level of professional experience (*P* < 0.001) (Table 3), with more experienced participants reporting higher levels of knowledge.

When participants were asked whether failure to maintain medical records could constitute a criminal offense under article 257/2 of the Turkish Penal Code, 67.2% responded correctly, 32.2% reported no opinion, and 0.6% responded incorrectly. No significant association was observed between this perceived adequacy of knowledge and professional experience (*P* =0.092) (Table 4).

In contrast, awareness of the legal consequences of falsifying or altering medical records was high, with 94.9% of participants responding correctly. This knowledge was significantly associated with professional experience (*P*=0.022); lower levels of experience were associated with higher rates of uncertainty or incorrect responses (Table 5).

Participants also demonstrated marked variability in recognising the statutory framework governing physicians’ professional responsibilities (Figure 5). Awareness was relatively high regarding commonly encountered legislation, including the Civil Servants Law (Law No. 657), the Turkish Penal Code, the Regulation on Inpatient Treatment Institutions, the Patient Rights Regulation, and the Medical Deontology Regulation. In contrast, recognition of several other laws defining physicians’ professional responsibilities was considerably lower, particularly for the Criminal Procedure Code, the Law on Private Hospitals, the Turkish Code of Obligations, the Military Penal Code, and the Law on the Socialization of Health Services.

## Discussion

An important finding of the present study was the discrepancy between participants’ self-reported medico-legal knowledge and their knowledge, assessed across multiple questionnaire domains. This finding highlights that self-perceived familiarity with medico-legal issues does not necessarily correspond to questionnaire-assessed factual knowledge.

The observed disparity between clinical proficiency and medico-legal awareness is consistent with previous literature indicating that physicians’ legal literacy frequently lags behind their technical and clinical expertise. Increasingly complex healthcare systems, expanding patients’ rights, and evolving legal frameworks require physicians not only to provide scientifically appropriate care but also to understand the legal and ethical responsibilities that accompany clinical decision-making. Importantly, malpractice risk is influenced not only by technical performance but also by communication, documentation quality, informed consent practices, organisational factors, and system-level processes. Studies evaluating malpractice risk and defensive medicine have similarly demonstrated that legal concerns substantially influence physicians’ professional behaviour and clinical decision-making.^9, 10, 14^ The present findings are also consistent with decades of evidence generated by the American Society of Anesthesiologists (ASA) Closed Claims Project. One of the major contributions of the Closed Claims Project has been the demonstration that serious anaesthesia-related injuries are frequently associated not only with technical failures but also with communication problems, documentation deficiencies, errors in clinical judgment, and system-related factors. Importantly, detailed medico-legal analyses have highlighted that understanding professional responsibilities and maintaining high standards of documentation and communication are essential components of safe anesthetic practice and effective risk management. These observations support the interpretation that strengthening medico-legal awareness should be regarded as an integral component of professional competence rather than merely a legal obligation. Anaesthesiology represents a unique context in this regard because perioperative care requires simultaneous responsibility for patient safety, interdisciplinary communication, documentation, equipment management, and rapid decision-making under time-critical conditions. The present findings are particularly relevant because approximately half of participants demonstrated insufficient awareness of operating room management and sterilisation responsibilities, while fewer than half correctly identified their responsibility for anaesthesia-related equipment and supplies. These findings warrant particular attention because equipment-related failures and organisational deficiencies continue to contribute to anaesthesia-related adverse events and malpractice litigation.^29^

An equally important observation concerns informed consent and physicians’ broader professional responsibilities. Consequently, deficiencies in physicians’ medico-legal awareness may extend beyond litigation risk and adversely influence physician-patient communication, trust, and the quality of perioperative care.^7^

Documentation represents another cornerstone of contemporary anaesthesiology practice and deserves particular emphasis when interpreting the present findings. Accurate perioperative documentation supports continuity of care, facilitates multidisciplinary communication, enables quality improvement initiatives, and provides an essential legal record in the event of malpractice investigations or judicial review. Comprehensive anaesthesia records are widely recognised as an integral component of safe perioperative practice and professional accountability. Therefore, the relatively limited awareness of documentation-related responsibilities should not be interpreted solely as a legal concern but also as an important quality-of-care issue directly relevant to perioperative patient management.^29, 30, 31^

The present findings should also be interpreted in the context of patient safety culture within modern anaesthesiology. The Helsinki Declaration on Patient Safety in Anaesthesiology emphasises that safe perioperative care depends not only on technical competence but also on effective communication, organisational responsibility, education, standardisation of practice, and continuous quality improvement. From this perspective, medico-legal competence should not be viewed merely as protection against litigation but rather as an integral component of patient safety systems. Improving physicians’ understanding of informed consent, documentation standards, institutional responsibilities, and professional accountability may, therefore, contribute to safer perioperative care through enhanced communication, transparency, and organisational performance, rather than through legal risk reduction alone.^31^

Our findings are also consistent with recent evidence from Türkiye analyzing closed anaesthesia malpractice claims between 2009 and 2022. That study demonstrated that perioperative mortality, intraoperative complications, delayed diagnosis, communication failures, and allegations of negligence continue to represent substantial medico-legal challenges within anaesthesia practice. Importantly, many of these events involve factors extending beyond technical proficiency alone, highlighting the importance of documentation quality, communication processes, organisational responsibility, and familiarity with applicable legal frameworks. The convergence of national malpractice data and the present survey findings further supports the need to strengthen medico-legal competence among practicing anaesthesiologists.^32^

The educational implications of these findings are considerable. Nearly half of physicians participating in the study self-reported no prior involvement in medico-legal educational activities despite substantial exposure to legal complaints during their professional careers. This observation suggests that existing educational frameworks may inadequately prepare physicians for the legal dimensions of contemporary clinical practice. Rather than relying exclusively on traditional lectures, residency programmes and continuing professional development initiatives may benefit from structured medico-legal curricula incorporating case-based learning, simulation exercises, documentation workshops, informed consent scenarios, interdisciplinary seminars involving legal experts, and periodic micro-learning modules. Professional organisations such as the TARD may play an important role in promoting such educational initiatives at a national level. Importantly, the educational value of closed claims analyses extends beyond litigation itself. By systematically examining rare but severe adverse events, closed claims reviews function as specialty-wide learning opportunities that enhance situational awareness, strengthen professional judgment, and improve physicians’ preparedness for uncommon but high-consequence clinical scenarios.

The relatively limited awareness of several key legal statutes, particularly the Turkish Code of Obligations and the Criminal Procedure Code, suggests that physicians are generally more familiar with regulations encountered in daily clinical practice than with broader medico-legal legislation that may nevertheless become highly relevant during judicial proceedings.

Taken together, the present findings suggest that strengthening medico-legal competence should be regarded not merely as a strategy for reducing malpractice litigation but as an essential component of professional accountability, effective communication, high-quality documentation, and safe anaesthesiology practice. Consistent with lessons learned from the ASA Closed Claims Project and the principles of the Declaration of Helsinki, improving medico-legal literacy should be considered an investment in patient safety, professional excellence, and continuous quality improvement.

### Study Limitations

This study has several important strengths. To our knowledge, this is among the first nationwide surveys in Türkiye to systematically examine professional and medico-legal awareness among anaesthesiology and reanimation physicians using a structured questionnaire. The relatively large sample size and inclusion of physicians with varying levels of professional experience provide a broad overview of current awareness levels at the national level. In addition, the multidimensional assessment of medico-legal awareness, including knowledge, education, legal exposure, and disciplinary awareness, allows for a more integrated evaluation of professional preparedness. Nevertheless, the nationwide distribution of the survey through the national professional society enabled participation by physicians working in various institutions and at different career stages, providing valuable insight into current medico-legal knowledge across a broad professional spectrum.

However, certain limitations should be acknowledged. The electronic distribution of the questionnaire through email and whatsapp may have introduced selection bias by preferentially reaching physicians who were more digitally engaged or interested in medico-legal issues. In addition, participation was voluntary, potentially introducing self-selection bias. Because the exact number of physicians who received or opened the survey could not be determined, the response rate could not be calculated. Therefore, although the survey included physicians from across Türkiye, the findings should not be interpreted as a statistically representative sample of all anaesthesiology and reanimation physicians in the country. Second, the cross-sectional design precludes causal inferences regarding the relationship between education, professional experience, and medico-legal awareness. The assessment of knowledge was based on questionnaire responses rather than objective testing; questionnaire responses may not fully reflect actual competency. Another limitation is that the questionnaire was specifically developed for this study and did not undergo formal psychometric validation or pilot testing. Therefore, the findings should be interpreted as reflecting physicians’ knowledge of the Turkish medico-legal framework rather than representing a universally validated measure of medico-legal competence. Although subgroup analyses according to professional experience, gender, and academic title were conducted, the sample size calculation was based on descriptive survey estimation methods rather than on statistical power for between-group comparisons. Consequently, subgroup findings should be interpreted as exploratory, particularly for categories with relatively small sample sizes. Although no direct personal identifiers were collected, the dataset was screened for potential duplicate submissions by reviewing demographic characteristics and response patterns before analysis. Future studies evaluating the impact of structured medico-legal training interventions on objective knowledge outcomes are warranted.

## Conclusion

Despite high awareness of core clinical responsibilities, participants demonstrated significant deficiencies in questionnaire-assessed knowledge across several medico-legal domains, and self-perceived knowledge of current legal regulations also remained limited. The findings highlight a significant gap between physicians’ real-world exposure to legal challenges and their perceived preparedness to navigate the increasingly complex medico-legal environment of contemporary healthcare.

Strengthening medico-legal competence should not be viewed solely as a strategy for reducing malpractice risk, but rather as an essential component of professional accountability, effective physician-patient communication, high-quality documentation, and safe anaesthesiology practice. Integrating structured medico-legal education into residency training and continuing professional development programmes may enhance physicians’ legal literacy and preparedness while supporting ethically responsible, evidence-based, and patient-centred clinical decision-making.

Ultimately, improving medico-legal awareness should be regarded as an essential component of professional excellence, patient safety, and high-quality anaesthesiology practice.

## Ethics

**Ethics Committee Approval:** This study was approved by the Non-Interventional Clinical Research Ethics Committee of Van Yüzüncü Yıl University Faculty of Medicine (Approval date: December 8, 2023; Decision No: 2023/13-10) and conducted in accordance with the Declaration of Helsinki.

**Informed Consent:** Written informed consent was obtained from all enrolled participants.

**Previous Presentation**

This study was presented as an oral presentation at the 59^th^ National TARK Congress, Antalya, Türkiye, December 4-7, 2025.

## Supplementary Materials

Supplementary Table https://d2v96fxpocvxx.cloudfront.net/66b874bd-7aaa-4f61-9199-52f558d61c0d/documents/2588.xlsx

## Figures and Tables

**Figure 1:**
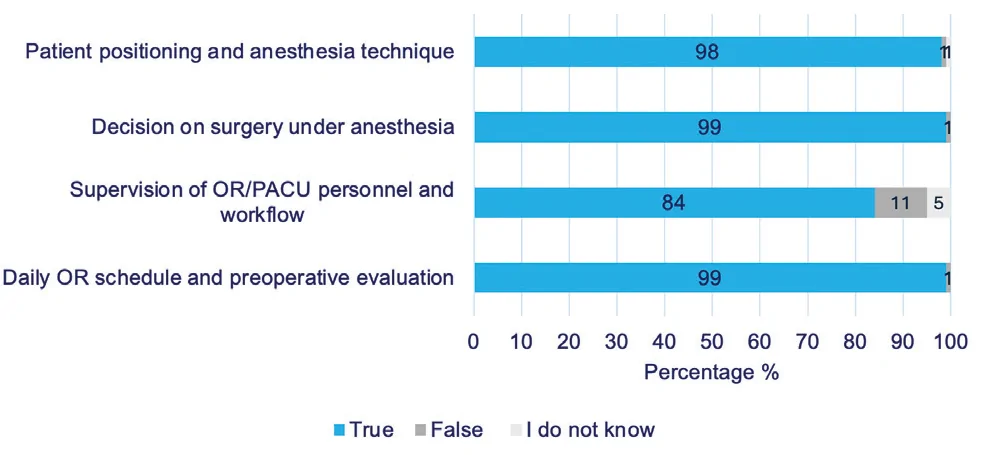
Questionnaire-assessed knowledge of core clinical responsibilities. Data are presented as percentages of responses (True/False/I do not know); n = 311. OR, operating room; PACU, postanesthesia care unit.

**Figure 2:**
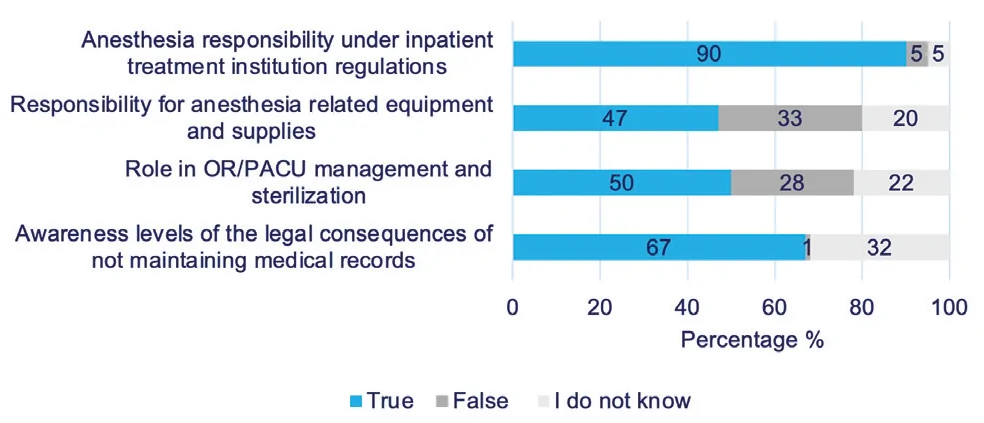
Questionnaire-assessed awareness of organisational responsibilities. Data are presented as percentages of responses (True/False/I do not know); n = 311. OR, operating room; PACU, postanesthesia care unit.

**Figure 3:**
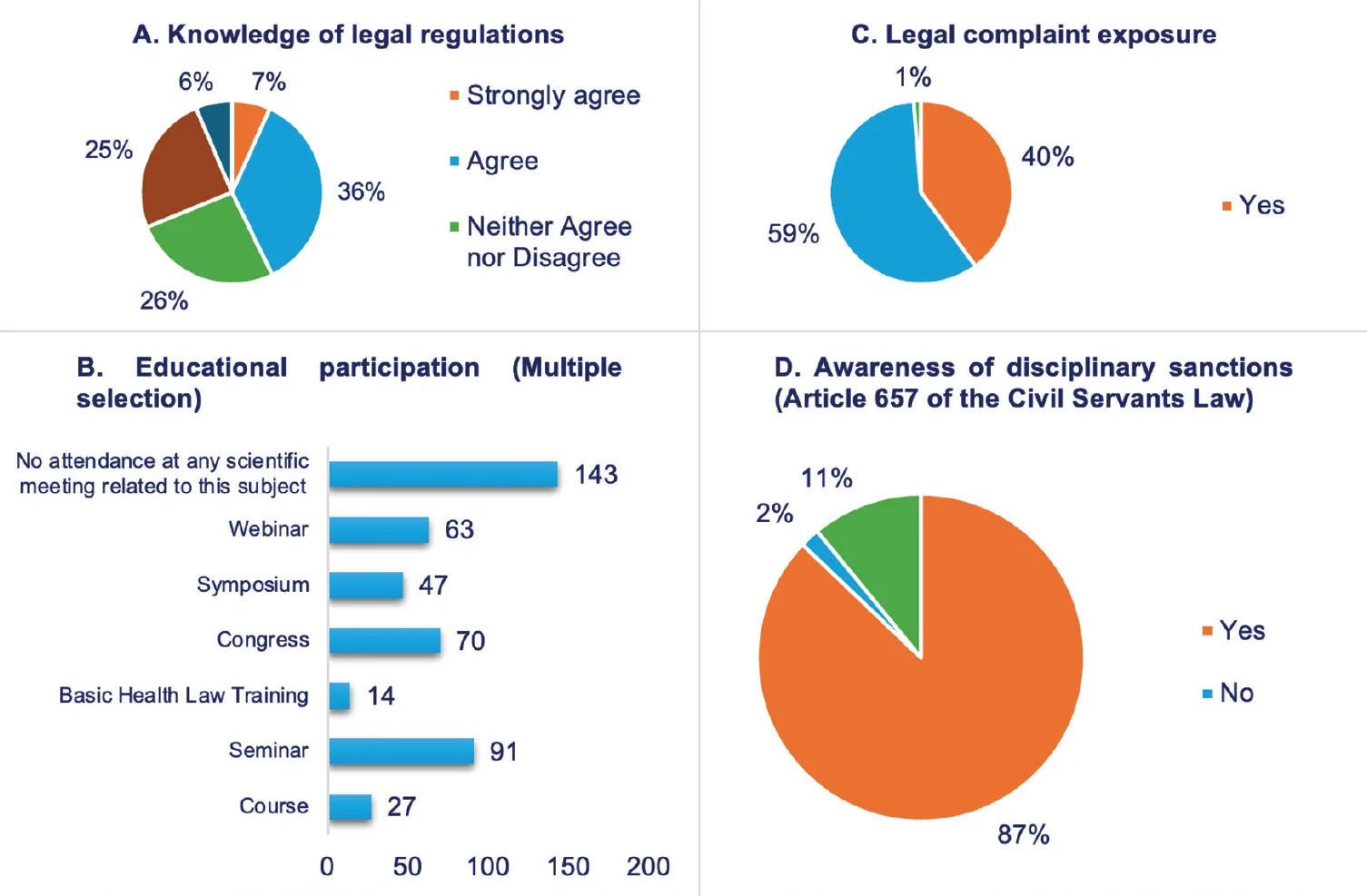
Self-reported medico-legal awareness and prior medico-legal exposure. Data are presented as number or percentages of responses; variability was observed across different domains. (A) Self-reported knowledge of legal regulations. (B) Participation in medico-legal educational activities (Multiple selection). (C) Exposure to legal complaints or judicial investigations. (D) Awareness of disciplinary sanctions under national regulations (n = 311).

**Figure 4:**
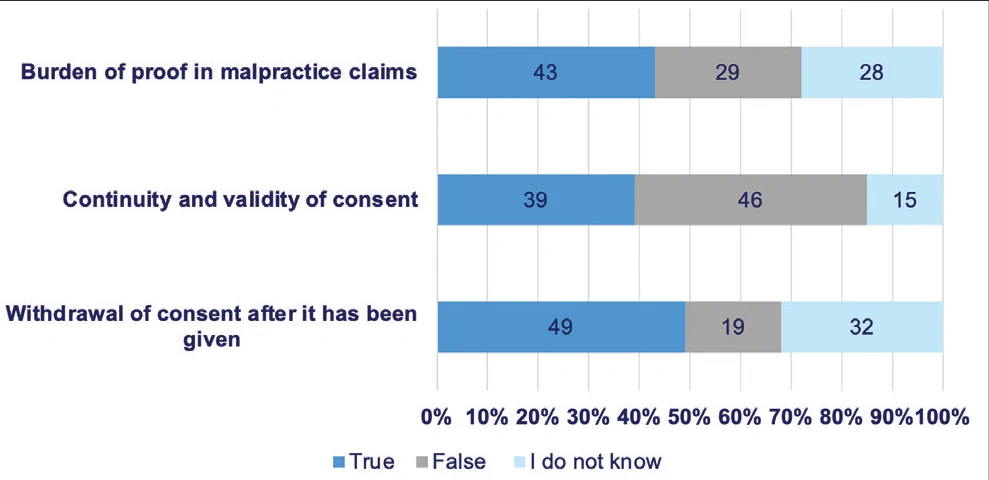
Questionnaire-assessed knowledge of informed consent and related legal principles. Data are presented as percentages of responses (True/False/I do not know); n = 311.

**Figure 5:**
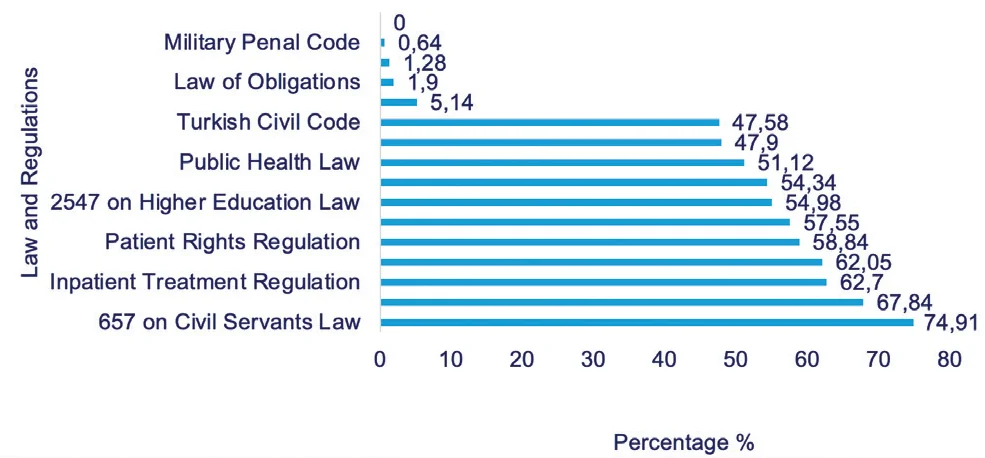
Percentage of participants correctly identifying laws and regulations relevant to physicians’ professional responsibilities. Data are presented as the percentage of participants who correctly identified each legal regulation; n = 311.

**Table 1. Structure and Content of the Survey Questionnaire:** 

**Section 1-Demographic parameters**
Age, sex, academic title, type of institution, duration of professional experience in anaesthesiology (years), province of practice
**Section** **2-Duties** **and** **authority** **of** **the** **anaesthesiologist**
The anaesthesiologist prepares the daily operating room work schedule. The anaesthesiologist performs the preoperative evaluation and premedication of patients scheduled for surgery. The anaesthesiologist supervises personnel working in the operating room and postoperative care units, ensures regular and efficient workflow and in-service training, and is responsible to the chief physician within the department. Depending on the patient’s health condition, the anaesthesiologist decides whether surgery may be performed under anaesthesia. The anaesthesiologist determines the most appropriate position on the operating table and selects the anaesthesia technique; administers the anaesthetic agent or ensures its administration under supervision. The anaesthesiologist is legally obligated to document the anaesthesia method and perioperative follow-up in the anaesthesia observation (record) form. The anaesthesiologist assists the operating room supervisor in operating room management and sterilization procedures. The anaesthesiologist is responsible for the provision, maintenance, preservation, and use of all anaesthesia-related devices, instruments, medications, and medical supplies in the operating room and postoperative care unit. According to the Regulation on the Operation of Inpatient Treatment Institutions, the anaesthesiologist is responsible for the anaesthesia administered to the patient.
**Section** **3-Legal** **responsibilities** **and** **applicable** **laws,** **regulations,** **and** **statutes**
I have comprehensive knowledge of the current laws, regulations, and statutes governing the legal responsibilities of anaesthesiologists in professional practice. Please specify the scientific meeting(s) you have previously attended regarding the professional and legal responsibilities of anaesthesiologists. Have you ever been subject to a legal complaint or judicial investigation related to healthcare services you provided in your professional practice? Which laws, statutes, and regulations currently in force define the legal framework of our professional responsibilities? (Multiple selection) Anaesthesiologists may incur legal liability for intentional or negligent acts arising from healthcare services or scientific research activities. Which of the following acts are classified as criminal offenses under the Turkish Penal Code? (Multiple selection) I am aware of the disciplinary sanctions that may be imposed following an investigation conducted under Article 657 of the Civil Servants Law and its related Disciplinary Regulations.
**Section** **4-Information** **disclosure,** **informed** **consent,** **and** **medical** **record-keeping** **obligations**
I understand that information disclosure and informed consent are not the same and that consent must be obtained in written form when legally required. Informed disclosure: prior to any planned medical intervention, the anaesthesiologist performing the procedure must provide the patient with the necessary information. The primary purpose is to eliminate the patient’s lack of knowledge, and the information may be provided verbally or in writing. Please select the mandatory elements that must be included in the informed disclosure process. (Multiple selection) The obligation to obtain patient consent is assigned to physicians under the Patient Rights Regulation and the Medical Deontology Regulation. Informed consent is defined as a person’s voluntary and informed acceptance of a medical intervention. No intervention violating bodily integrity or personal rights may be performed without consent. A reasonable period must be granted for the patient to consider providing consent. What is the minimum required time? (This item was excluded from the interpretation of questionnaire-assessed factual knowledge because the applicable regulation does not establish a universally applicable 24-hour minimum timeframe.) Consent must be present at the beginning of treatment and remain valid throughout its duration. Except for legal exceptions, consent is not bound to a specific form and may be explicit or implicit, written or verbal. Consent may be withdrawn after being given. I understand the legal and procedural implications of a patient withdrawing consent and the appropriate course of action in such cases. In medical malpractice claims, the burden of proof lies with the physician to demonstrate absence of fault. Please specify the physician’s documentation obligations and potential criminal liabilities related to medical records. (Multiple selection) In anaesthesia monitoring records, any crossed-out or corrected entries must remain legible. Who holds ownership of medical records, including anaesthesia monitoring forms? For how many years must anaesthesia records (anaesthesia monitoring forms) be retained at a minimum? Altering medical records or creating false documentation may constitute the criminal offense of forgery of private or official documents under the law. Since public-sector physicians have a legal obligation to maintain medical records, failure to do so may constitute a criminal offense under Article 257/2 of the Turkish Penal Code. I would like to attend a training program on the professional and legal responsibilities of anaesthesiologists. Abbreviations and Response Formats: Likert-type items were rated on a 5-point scale (1= Strongly disagree, 2= Disagree, 3= Neither agree nor disagree, 4= Agree, 5= Strongly agree). For knowledge-based items, response options were structured as True/False/I do not know or Yes/No/I do not know, as appropriate. Multiple-selection questions allowed participants to select more than one response option where applicable.

**Table 2. Demographic and Professional Characteristics of the Participants:** 

**Variable**	**n**	**%**
**Gender**		
Male	184	59.2
Female	127	40.8
**Academic** **title**		
Professor	15	4.8
Associate Professor	19	6.1
Assistant Professor	18	5.8
Lecturer	7	2.3
Specialist Physician	164	52.7
Resident	88	28.3
**Professional** **experience** **(year)**		
0-5	74	23.8
6-10	84	27.0
11-20	100	32.2
>20	53	17.0
**Institution** **type**		
State University Hospital	120	38.6
Foundation University Hospital	4	1.3
Training and Research Hospital	59	19.0
Affiliated/Contracted Hospitals	17	5.5
City Hospital	17	5.5
State Hospital	55	17.7
Private Hospital	39	12.5

**Table 3. Self-Reported Knowledge of Legal Regulations According to Professional Experience:** 

**Professional experience (years)**	**Strongly agree**	**Agree**	**Neither agree nor disagree**	**Disagree**	**Strongly** **disagree**	**Total**	***P *value**
0-5	4	16	27	19	8	74	<0.001^χ2^
6-10	3	26	28	21	6	84	
11-20	5	37	21	32	5	100	
>20	9	33	5	5	1	53	
Total	21	112	81	77	20	311	

χ^2^: Chi-square test; Fisher’s exact test when appropriate

**Table 4. Awareness of Legal Responsibility for Failure to Maintain Medical Records According to Professional Experience:** 

**Professional experience (years)**	**True (n)**	**False (n)**	**I do not know (n)**	**Total (n)**	***P* value**
0-5	56	0	18	74	0.092^χ2^
6-10	57	0	27	84	
11-20	62	0	38	100	
>20	34	2	17	53	
Total (n)	209	2	100	311	-

χ^2^: Chi-square test; Fisher’s exact test when appropriate

**Table 5. Awareness of Legal Consequences of Falsifying Medical Records According to Professional Experience:** 

**Professional experience (years)**	**True (n)**	**False (n)**	**I do not know (n)**	**Total (n)**	***P* value**
0-5	64	1	9	74	0.022^ χ2^
6-10	82	0	2	84	
11-20	98	0	2	100	
>20	51	1	1	53	
Total (n)	295	2	14	311	-

χ^2^: Chi-square test; Fisher’s exact test when appropriate
